# Calmodulin Antagonist W-7 Enhances Intermediate Conductance Ca^2+^- Sensitive Basolateral Potassium Channel (IK_Ca_) Activity in Human Colonic Crypts

**DOI:** 10.1007/s00232-021-00193-y

**Published:** 2021-07-27

**Authors:** Kate A. Bowley, Geoffrey I. Sandle

**Affiliations:** Leeds Institute of Medical Research at St James, St James University Hospital, Beckett Street, Leeds, LS9 7TF UK

**Keywords:** Calmodulin, Colon, Potassium channels, W-7

## Abstract

Intermediate conductance potassium (IK_Ca_) channels are exquisitively Ca^2+^ sensitive, intracellular Ca^2+^ regulating channel activity by complexing with calmodulin (CaM), which is bound to the cytosolic carboxyl tail. Although CaM antagonists might be expected to decrease IK_Ca_ channel activity, the effect of W-7 in human T lymphocytes are conflicting. We therefore evaluated the effect of W-7 on basolateral IK_Ca_ channels in human colonic crypt cells. Intact crypts obtained from normal human colonic biopsies by Ca^2+^ chelation were used for patch clamp studies of basolateral IK_Ca_ channels in the cell-attached configuration. IK_Ca_ channel activity was studied when the bath Ca^2+^ concentration was changed from 1.2 mmol/L to 100 μmol/L and back to 1.2 mmol/L, as well as from 100 μmol/L to 1.2 mmol/L and back to 100 μmol/L, both in the absence and presence of 25 μmol/L W-7. Decreasing bath Ca^2+^ from 1.2 mmol/L to 100 μmol/L decreased IK_Ca_ channel activity reversibly in the absence of W-7, whereas there was a uniformly high level of channel activity at both bath Ca^2+^ concentrations in the presence of W-7. In separate experiments, increasing bath Ca^2+^ from 100 μmol/L to 1.2 mmol/L increased IK_Ca_ channel activity reversibly in the absence of W-7, whereas there was again a uniformly high level of channel activity at both bath Ca^2+^ concentrations in the presence of W-7. We, therefore, propose that W-7 has a specific stimulatory effect on basolateral IK_Ca_ channel activity, despite its ability to inhibit Ca^2+^/CaM-mediated, IK_Ca_ channel-dependent Cl^−^ secretion in human colonic epithelial cells.

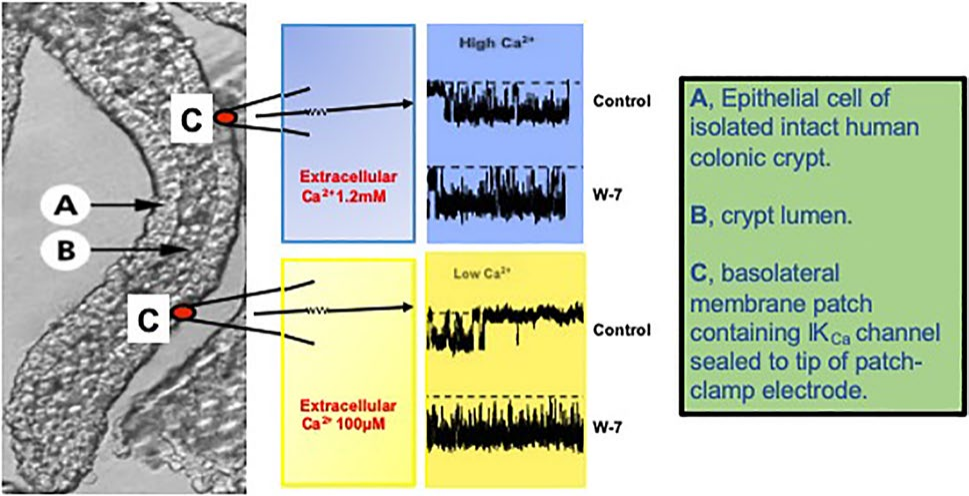

## Introduction

Intermediate conductance Ca^2+^-sensitive K^+^ channels (IK_Ca_) dominate the K^+^ conductance of the basolateral membrane in human colonic crypts (Al-Hazza et al. 2012). These K^+^ channels are activated by increases in intracellular Ca^2+^ and cAMP, and rapidly inhibited in a non-genomic manner by aldosterone (Sandle et al. 1994; Bowley et al. 2003). They are a major component of the electrogenic Cl^−^ secretory process in human colon and readily inhibited by somatostatin and its stable synthetic analogue octreotide, which is widely used as an anti-diarrhoeal agent (Sandle et al. 1999). Their inhibitory effects on human colonic IK_Ca_ channels are mediated by inhibitory G proteins and may reflect a decrease in the sensitivity of the channels to intracellular Ca^2+^ (Sandle et al. 1999). IK_Ca_ channels are exquisitively sensitive to Ca^2+^, despite lacking Ca^2+^-binding sites such as ‘EF-hand’ and ‘Ca^2+^ bowl’ motifs in their sequences (Joiner et al. 1997; Ishii et al. 1997; Logsdon et al. 1997). The high affinity of IK_Ca_ channels for Ca^2+^ reflects the constitutive Ca^2+^-independent interaction of one calmodulin (CaM) molecule with the cytosolic COOH terminus of each channel subunit (Fanger et al. 1999; Khanna et al. 1999). Under these circumstances, Ca^2+^ binding to CaM induces a conformational change in CaM, which is transmitted to the channel subunits, leading to channel gating (Fanger et al. 1999).

CaM antagonists such as W-7, trifluoperazine and calmidazolium prevent interactions between CaM and target proteins by reversibly binding to the hydrophobic areas of CaM exposed after Ca^2+^ binding (Ikura 1996). Studies into the interaction between CaM and IK_Ca_ channel subunits have produced conflicting results, CaM antagonists either inhibiting or having no effect on IK_Ca_ channel activity (Fanger et al. 1999; Khanna et al. 1999; del Carlo et al. 2002). In this study, the initial aim was to investigate the role of CaM in Ca^2+^-regulated gating of basolateral IK_Ca_ channels in human colonic crypt cells using the CaM antagonist W-7, which inhibits IK_Ca_ channel currents in human T lymphocytes (Fanger et al. 1999), but at the outset it became clear that W-7 itself had a marked stimulatory effect on IK_Ca_ channels in human colonocytes.

## Methods

### Isolation of Human Colonic Crypts

After obtaining written informed consent, 4–6 biopsies of normal-looking sigmoid colonic mucosa were taken during colonoscopy or flexible sigmoidoscopy in patients being investigated for altered bowel habit. None of the patients were receiving medication at the time of the procedure. Patients undergoing colonoscopy self-administered Klean-Prep® (Norgine) during the preceding 24 h, and those undergoing flexible sigmoidoscopy received a Fleet® ready-to-use enema (De Witt) before the procedure. Routine histology was normal in all cases. The study was approved by the Leeds Health Authority Ethics Committee. Intact crypts were isolated by a modification of a previously described Ca^2+^ chelation technique (Bowley et al. 2003), suspended in a storage solution containing (mmol/L): 100 K^+^ gluconate, 30 KCl, 20 NaCl, either 1.2 or 0.1 CaCl_2_, 1 MgCl_2_, 10 HEPES, 5 glucose, 5 Na^+^ pyruvate, 5 Na^+^ butyrate, supplemented with 1 g/L bovine serum albumin, titrated to pH 7.4 with KOH, and kept on ice until required.

### Patch Clamp Studies

Recordings were obtained from basolateral membrane patches of cells in the middle third of crypts in the cell-attached configuration. Experiments were performed at 20–22 °C rather than 37 °C to maintain cell viability (Sträter et al. 1996). The bath solution contained (mmol/L): 140 NaCl, 4.5 KCl, either 1.2 or 0.1 CaCl_2_, 1.2 MgCl_2_, 5 glucose, 10 HEPES, 5 Na^+^ butyrate, and titrated to pH 7.4 with NaOH. Patch pipettes fabricated from borosilicate glass were filled with a solution containing (mmol/L): 145 KCl, 1.2 CaCl_2_, 1.2 MgCl_2_, 5 glucose, 10 HEPES, 5 Na^+^ butyrate, and had resistances of 5-6MΩ. Single channel currents were recorded with a patch clamp amplifier (List model EPC7, Darmstadt, Germany) at a holding voltage of − 40 mV, referenced to the pipette interior. Currents were stored on videotape after pulse code modulation (PCM-701 ES, Sony, Japan). Stored currents were low pass filtered (750 Hz) and loaded into computer memory via a DigiData 1200 interface system using pClamp software (version 5.6, Axon Instruments Inc, USA) for off-line analysis. Single channel open probability (*P*_O_) was calculated using an analysis programme written in Quick Basic 4.0 (Microsoft, USA), as previously described (Lomax et al. 1996).

All recordings were made in the cell-attached configuration, while crypts were superfused continuously with NaCl solution containing either 1.2 mmol/L or 100 μmol/L unbuffered Ca^2+^. The potential role of CaM in regulating basolateral IK_Ca_ channel activity was evaluated using the CaM antagonist W-7 at both bath Ca^2+^ concentrations. Crypts bathed initially in 100 μmol/L Ca^2+^ were stored in a K^+^-rich solution containing 100 μmol/L CaCl_2_ rather than the usual 1.2 mM CaCl_2_. To determine the effect of W-7, crypts were pre-incubated with 25 μmol/L W-7 for 20 min, and continuously exposed to this concentration throughout the experiment.

### Statistical Analysis

Results are expressed as mean ± standard error (SE). Statistical analyses were performed using the Student’s *t* test, where *P* < 0.05 was considered significant.

## Results

In preliminary experiments, 25 μmol/L W-7 had no effect on channel activity at a bath Ca^2+^ concentration of 1.2 mmol/L, However, W-7 stimulated channel activity at a bath Ca^2+^ concentration of 100 μmol/L (data not shown). Subsequent experiments were, therefore, done to test the effect of W-7 when the bath Ca^2+^ concentration was switched from 1.2 mmol/L to 100 μmol/L, and vice versa.

### ***Effect of W-7 on IK***_***Ca***_*** Channel Activity in Response to Lowering Bath Ca***^***2***+^

**Fig. 1:**
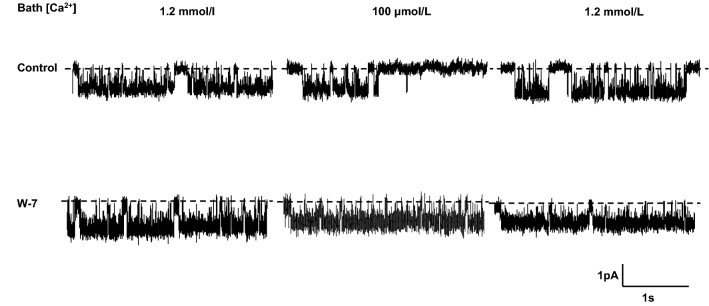
IK_Ca_ channel activity when bath Ca^2+^ concentration changed from 1.2 mmol/L to 100 μmol/L, and back to 1.2 mmol/L in the absence (control) and presence of W-7

Representative recordings showing the effect of W-7 on the response of IK_Ca_ channel activity to lowering bath Ca^2+^ from 1.2 mmol/L to 100 μmol/L are shown in Fig. 1. In the absence of W-7 (control), there was a high level of IK_Ca_ channel activity when the bath solution initially contained 1.2 mmol/L Ca^2+.^ Within 2 min of lowering bath Ca^2+^ to 100 μmol/L, there was a sustained decrease in channel activity, consistent with a decrease in intracellular Ca^2+^ concentration. Channel activity was restored to its original high level by raising bath Ca^2+^ to 1.2 mmol/L. When this protocol was repeated in the presence of 25 μmol/L W-7, the high level of channel activity at a bath concentration of 1.2 mmol/L was similar to that in the absence of W-7. However, a striking finding was that in the presence of W-7, lowering bath Ca^2+^ concentration to 100 μmol/L failed to decrease channel activity, and the subsequent raising of bath Ca^2+^ to 1.2 mmol/L had no additional effect.

Data obtained from experiments when bath Ca^2+^ was changed from 1.2 mmol/L to 100 μmol/L and then back to 1.2 mmol/L, either in the absence of W-7 (controls, *n* = 5), or in the presence of W-7 (*n* = 6), are summarized in Table 1. W-7 had no effect on IK_Ca_ channel activity at a bath Ca^2+^ concentration of 1.2 mmol/L. Whereas channel activity decreased by 49% (*P* < 0.005) when bath Ca^2+^ was lowered from 1.2 mmol/L to 100 μmol/L in the absence of W-7, there was no change in channel activity when bath Ca^2+^ was lowered in the presence of W-7. These data suggest that W-7 may have ‘clamped’ IK_Ca_ channel activity at a level (which was dependent on the initial intracellular Ca^2+^ concentration) when the bath solution contained 1.2 mmol/L Ca^2+^. To explore this possibility, additional experiments were done at an initial bath Ca^2+^ concentration of 100 μmol/L.

**Table 1:** Effect of W-7 on single IK_Ca_ channel open probability (*P*_O_) at high initial bath Ca^2+^ concentration

Bath Ca^2+^ concentration			
	1.2 mmol/L	100 μmol/L	1.2 mmol/L
Control (*n* = 5)	0.612 ± 0.064	0.321 ± 0.087	0.500 ± 0.095
W-7 (*n* = 6)	0.645 ± 0.073	0.700 ± 0.042	0.646 ± 0.084
*P*	N.S	< 0.003	N.S

### ***Effect of W-7 on IK***_***Ca***_*** Channel Activity in Response to Raising Bath Ca***^***2***+^

**Fig. 2:**
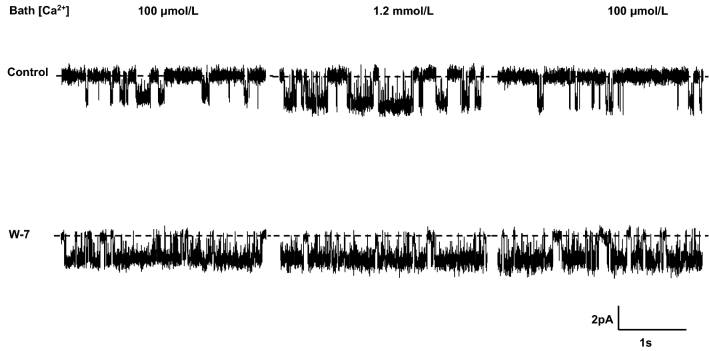
IK_Ca_ channel activity when bath Ca^2+^ concentration changed from 100 μmol/L to 1.2 mmol/L, and back to 100 μmol/L in the absence (control) and presence of W-7

Representative recordings showing the effect of W-7 on the response of IK_Ca_ channel activity to raising bath Ca^2+^ from 100 μmol/L to 1.2 mmol/L are shown in Fig. 2. In the absence of W-7 (control) there was a relatively low level of IK_Ca_ channel activity when the bath solution initially contained 100 μmol/L. Within 2 min of raising bath Ca^2+^ to 1.2 mmol/L, there was a marked increase in channel activity, consistent with an increase in intracellular Ca^2+^ concentration, and channel activity decreased to the basal level when bath Ca^2+^ was lowered to 100 μmol/L. Repeating this protocol in the presence of W-7, the high level of channel activity at an initial bath Ca^2+^ concentration of 100 μmol/L was similar to that seen at an initial bath Ca^2+^ concentration of 1.2 mmol/L in the absence of W-7. Channel activity did not increase further when bath Ca^2+^ concentration was raised to 1.2 mmol/L, and was unchanged when bath Ca^2+^ was subsequently lowered to 100 μmol/L.

Data obtained from experiments when bath Ca^2+^ was changed from 100 μmol/L to 1.2 mmol/L and then back to 100 μmol/L, either in the absence of W-7 (controls, *n* = 6), or in the presence of W-7 (*n* = 4), are summarized in Table 2. W-7 elicited a greater than twofold increase in IK_Ca_ channel activity at a bath Ca^2+^ concentration of 100 μmol/L. Whereas channel activity increased by 84% (*P* < 0.001) on raising bath Ca^2+^ from 100 μmol/L to 1.2 mmol/L in the absence of W-7, channel activity was unaffected when the same change in bath Ca^2+^ was performed in the presence of W-7. Thus, these observations suggest that W-7 stimulates basolateral IK_Ca_ channels in human colonic crypt cells by a mechanism that appears to be largely (if not entirely) independent of the intracellular Ca^2+^ concentration, rather than by ‘clamping’ channel activity at a high level commensurate with the intracellular Ca^2+^ concentration that prevailed at a bath Ca^2+^ concentration of 1.2 mmol/L.

**Table 2:** Effect of W-7 on single IK_Ca_ channel open probability (*P*_O_) at low initial bath Ca^2+^ concentration

Bath Ca^2+^ concentration			
	100 μmol/L	1.2 mmol/L	100 μmol/L
Control (*n* = 6)	0.271 ± 0.080	0.499 ± 0.090	0.128 ± 0.049
W-7 (*n* = 4)	0.661 ± 0.073	0.703 ± 0.046	0.711 ± 0.053
*P*	< 0.01	N.S	< 0.0001

## Discussion

The initial aim of this study was to evaluate the effect of the CaM antagonist W-7 on the regulation of basolateral IK_Ca_ channels by Ca^2+^-mediated agonists in human colonic crypt cells. However, in preliminary experiments, rather than producing little or no change in basal channel activity (that is, in the absence of a Ca^2+^-mediated agonist), W-7 prevented the decrease in channel activity that occurred when bath Ca^2+^ concentration was lowered from 1.2 mmol/L to 100 μmol/L. We therefore focused on evaluating the effect of W-7 on human colonic crypt cells in the absence of Ca^2+^-mediated agonists. The data in Table 1 and Table 2 show that in the absence of W-7, decreasing or increasing extracellular Ca^2+^ concentration elicited the expected changes in IK_Ca_ channel activity, which presumably reflected corresponding decreases or increases in intracellular Ca^2+^ concentration. In the absence of W-7, *P*_O_ was 0.5–0.6 at a bath Ca^2+^ concentration of 1.2 mmol/L, and decreased by ~ 50% when bath Ca^2+^ was lowered to 100 μmol/L. By contrast, P_O_ was invariably higher (~ 0.7) in the presence of W-7, irrespective of the bath Ca^2+^ concentration. This novel and entirely unexpected observation suggested that amongst the CaM inhibitors, W-7 at least has the ability to stimulate IK_Ca_ channels, which raises questions about its suitability in studies designed to evaluate the role of CaM in IK_Ca_ channel regulation. Coincidentally, it may be that the level of basolateral IK_Ca_ channel activity seen in human colonic crypt cells in the presence of W-7 is the highest achievable, since previous studies have always indicated lower levels of activity, even after using thapsigargin to increase intracellular Ca^2+^ concentration (Sandle et al. 1999).

Unlike our results, previous studies have shown that CaM inhibitors such as W-7, trifluoperazine (TFP), and calmidazolium, either had no effect or inhibited the activity of native and cloned IK_Ca_ channels. Thus, W-7, TFP or calmidazolium had no effect on whole-cell IK_Ca_ currents at physiological membrane voltages in human T lymphocytes and KCNN4-transfected COS-7 cells (Fanger et al. 1999). Voltage-dependent inhibition of IK_Ca_ currents was seen in depolarized cells, probably reflecting a direct effect on the channel rather than via modulation of CaM activity (Fanger et al. 1999). The ineffectiveness of CaM antagonists at physiological voltages could reflect the constitutive nature of the binding between CaM and IK_Ca_ channels, which may prevent access of W-7 to its site of action during conformational changes in the N- and C-terminal domains of CaM triggered by Ca^2+^ binding to EF hands (Ikura 1996). CaM antagonists may therefore be unable to antagonize the interaction between the hydrophobic region of Ca^2+^-bound CaM and the IK_Ca_ channel CaM-binding domain in the proximal C terminus (Fanger et al. 1999).

Our finding that W-7 altered the response of basolateral K_Ca_ channels in human colonocytes to changes in bath Ca^2+^ concentration differs from studies in human erythrocytes, which showed that Ca^2+^-dependent IK_Ca_ channel activity in cell-attached and excised inside-out patches was unaffected by 10–100 μmol/L W-7 (del Carlo et al. 2002). CaM binding to IK_Ca_ channel α-subunits is thought to be Ca^2+^-independent, and the interaction between CaM and its binding domain on IK_Ca_ channel protein may occur early in channel biogenesis, before insertion into the cell membrane (Fanger et al. 1999). However, whole-cell IK_Ca_ currents in human T lymphocytes are inhibited by CaM antagonists and the CaM kinase antagonist KN-62, which suggests a role for CaM kinases in IK_Ca_ channel regulation (Khanna et al. 1999). In addition, cloned KCNN4 channels expressed in CHO cells were shown to be blocked by W-7 and TFP in a voltage-dependent manner, suggesting that these CaM antagonists may have inhibited channel activity by impeding the interaction of the hydrophobic region of CaM with IK_Ca_ channel protein (Fanger et al. 1999).

The results of the present study strongly suggest that W-7 has a direct stimulatory effect K_Ca_ channel activity in human colonocytes which is CaM-independent. Consistent with this view, W-7 (20–70 μmol/L) has previously been shown to increase ^86^Rb (K^+^) influx and efflux in the human salivary epithelial cells line HSG-PA in a partially Ca^2+^-dependent manner (Patton et al. 1991). The overall conclusion from those studies was that W-7 stimulated K^+^ fluxes in HSG-PA cells via a mechanism involving direct or indirect interaction with K^+^ channels in a way that differed from that seen during muscarinic (carbachol) stimulation or in response to the Ca^2+^ ionophore A23187 (Patton et al. 1991).

While the precise mechanism by which W-7 might interact with K^+^ channels remains to be established, one attractive possibility has come from studies using large unilamellar vesicles (LUVs) (Sengupta et al. 2007). These studies were based on the fact that the naphthalene-sulfonamide derivatives W-7 and W-13 are amphipathic weak bases. At concentrations where they may bind to the Ca^2+^/CaM complex by nonspecific hydrophobic and electrostatic interactions, they may also bind to the inner leaflet of the plasma membrane. This would have the effect of decreasing the inner leaflet’s net negative charge, which mainly reflects the presence of the monovalent acidic lipid 1-palmitoyl-2-oleoyl-*sn*-glycerol-3-phosphatidylserine (PS). Consistent with this view, the inclusion of PS produced a negative electrostatic potential in the aqueous phase close to the vesicle surface which decreased exponentially with distance, the zeta potentlal (*ζ*) being the electrostatic potential ~ 2 Å from the membrane surface, and the addition of 30 μmol/L W-7 to the LUVs decreased *ζ* by ~ 23 mV (Sengupta et al. 2007). We, therefore, speculate that if W-7 depolarized *ζ* at the inner leaflet of the colonocyte basolateral membrane, voltage-sensitive Ca^2+^ channels within the membrane may have been activated (Brice and Dolphin 1999), resulting in a rise in intracellular Ca^2+^ to a level sufficient to increase IK_Ca_ channel activity, even at a bath Ca^2+^ of 100 μmol/L. This offers a plausible explanation for our observation that IK_Ca_ channel activity at a bath Ca^2+^ concentration of 100 μmol/L in the presence of W-7 was similar to that at a bath Ca^2+^ concentration of 1.2 mmol/L in the absence of W-7. We also considered two other explanations. The first is that W-7-induced depolarization of *ζ* might have resulted in a purely voltage-dependent increase in IK_Ca_ channel activity but this is unlikely since basolateral IK_Ca_ channels in human colon are inherently voltage-insensitive (Bowley et al. 2003). The second, which also seems unlikely, is that W-7 enhanced the already exquisite Ca^2+^-sensitivity of IK_Ca_ channels to the extent that their activity at a bath Ca^2+^ of 100 μmol/L increased to the level seen at a bath Ca^2+^ concentration of 1.2 mmol/L. Thus, in studies where W-7 is used to investigate CaM’s role in Ca^2+^-mediated Cl^−^ secretion (specifically in human colonic epithelium), where basolateral IK_Ca_ channels are a critical component (Tabcharani et al. 1994; Duan et al. 2019), W-7 may actually upregulate these channels independently of CaM while inhibiting overall Cl^−^ secretion.
